# Use of a Mobile Application for Self-Monitoring Dietary Intake: Feasibility Test and an Intervention Study

**DOI:** 10.3390/nu9070748

**Published:** 2017-07-13

**Authors:** Ji-Eun Lee, Sihan Song, Jeong Sun Ahn, Yoonhee Kim, Jung Eun Lee

**Affiliations:** 1 Department of Food and Nutrition, Sookmyung Women’s University, Seoul 04310, Korea; jsilver1109@gmail.com; 2 Department of Food and Nutrition, College of Human Ecology, Seoul National University, Seoul 08826, Korea; songsihan@snu.ac.kr (S.S.); jungsunan@snu.ac.kr (J.S.A.); 3 Department of Computer Science, Sookmyung Women’s University, Seoul 04310, Korea; yulan@sookmyung.ac.kr

**Keywords:** mobile application, mobile health care, dietary assessment, feasibility, pre–post intervention

## Abstract

Given the increasing social and economic burden of chronic disease and the need for efficient approaches to prevent and treat chronic disease, emphasis on the use of information and communication technology (ICT)-based health care has emerged. We aimed to test the feasibility of a mobile application, Diet-A, and examine whether Diet-A could be used to monitor dietary intake among adolescents. In a three-month pre–post intervention study, 9 male and 24 female high school students aged 16–18 years consented and participated in this study. Participants were instructed to record all foods and beverages consumed using voice or text mode input. Nutrient intake was measured using 24-h recalls pre- and post-intervention. We compared nutrient intake data assessed by Diet-A application with those assessed by 24-h recalls. Participants tended to underreport intakes of nutrients compared to those assessed by two 24-h recalls. There were significant decreases in sodium (*p* = 0.04) and calcium (*p* = 0.03) intake between pre- and post-intervention. Of participants who completed questionnaires of feasibility (*n* = 24), 61.9% reported that they were satisfied using the application to monitor their food intake, and 47.7% liked getting personal information about their dietary intake from the application. However, more than 70% of participants answered that it was burdensome to use the application or that they had trouble remembering to record their food intake. The mobile application Diet-A offers the opportunity to monitor dietary intake through real-time feedback. However, use of Diet-A may not provide accurate information on the food intake of adolescents, partly because of the recording burden.

## 1. Introduction

The World Health Organization (WHO) reported that deaths caused by noncommunicable chronic diseases (NCD) are expected to increase from 36 million in 2010 to 44 million in 2020 [1]. The leading contributing factors to the global burden of disease in 2010 were high blood pressure, smoking, household air pollution, low fruit intake, and alcohol use [2], suggesting the importance of lifestyle in maintaining health. Given the increasing social and economic burden of chronic disease and the need for efficient approaches to prevent and treat chronic disease, emphasis on the implementation of information and communication technology (ICT)-based health care has emerged.

Advancements in technology have been expected to help health professionals and health care consumers teach and adopt healthy behaviors and prevent chronic disease in a cost-effective way. Diet is a major risk factor or prevention-related factor of noncommunicable chronic diseases including cancer, cardiovascular disease and diabetes. To prevent and control a noncommunicable chronic disease, the WHO established a strategy for healthy eating and physical activity and prepared a series of action plans [3], suggesting the importance of diet in maintaining a healthy life.

Assessment of dietary intake and intervention in dietary behaviors are key components in health care and public health. Accurate methods and tools to assess food and nutrient intake are essential in monitoring nutritional status. Twenty-four-hour dietary recall, dietary records, and food frequency questionnaires (FFQs) are commonly used to measure dietary intake [4]. Twenty-four-hour dietary recall can be administered by a trained interviewer, who asks the respondent for details of foods and beverages consumed within 24-h and records the information. Therefore, literacy of the respondent is not required, and the method can be used in a wide range of populations. However, this method depends on the respondent’s memory and requires trained interviewers. For dietary records, the respondent records all foods and beverages consumed over one or more days. Although dietary records are often regarded as a gold standard, participants need to be highly motivated and have the ability to record the details of their own diet. A FFQ asks respondents to report their usual frequency of consumption of a list of provided foods for designated time periods. This method is useful for examining usual dietary intake in a large population, but a FFQ cannot contain all foods and beverages that an individual consumed. Given such limitation of each method, the accuracy of dietary measurement for free-living individuals remains challenging in nutritional epidemiology research.

Health-related behaviors, such as healthy eating and regular exercise, could lead to improvement in population health only if they are sustained. Mobile technologies can help people record their food intake and monitor their diet in real time. The ENGAGED randomized controlled trial examined the efficacy of a smartphone-supported weight loss program among adults whose BMI was between 30 and 40 kg/m^2^ and found that self-guided intervention with use of a mobile phone could be a potential alternative to the face-to-face treatment [5]. In the ENGAGED trial, participants monitored their dietary intake using application of smartphone. Another weight loss program that implemented a mobile phone application “My Meal Mate” reported higher frequency of use and greater weight loss compared to a diary group or website group [6]. The convergence of information and communication technology (ICT) and nutrition may advance dietary assessment and help individuals obtain accurate and personalized data, leading to an improvement of their health through daily feedback.

According to research released in 2015, Korea (83%) has been ranked fourth among 56 countries in smartphone penetration, outranked only by the United Arab Emirates(UAE) (90.8%), Singapore (87.7%), and Saudi Arabia (86.1%) [7]. The International Telecommunication Union (ITU) reported that Korea ranked the first in the ITU’s ICT Development Index (IDI), a composite measurement reflecting level of ICT access, use and skill [8]. Korea has great potential to test and evaluate mobile health care technology, given its high rate of smartphone use and good quality of ICT infrastructure.

Therefore, we developed a self-monitoring mobile dietary application, Diet-A, and tested its feasibility in a pre–post intervention study. We also aimed to examine whether the mobile application could be a useful tool for adolescents to monitor their dietary intake.

## 2. Materials and Methods 

### 2.1. Development of the Mobile Application

**Figure 1:**
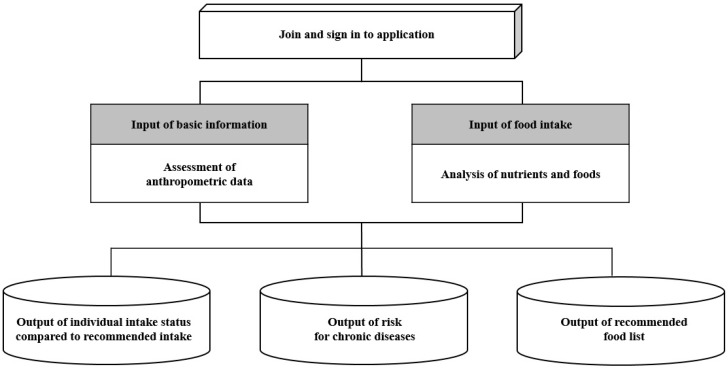
Flow chart of developing stage of application.

We developed a dietary self-monitoring mobile application called ‘Diet-A’; its development process has been published elsewhere [9,10]. The menu of the application was composed of three parts: records of dietary intake, real-time feedback, and provision of information on disease prevention in accordance with the participant’s input (Figure 1). The structure of the Diet-A system allows input of dietary data, calculation of nutrients, comparison with dietary reference intake, production of descriptive statistics of nutrient intakes, and display of personalized advice, and a food recommendation list [9]. After users logged into Diet-A, they entered their sex, age, height and weight, and then their estimated energy requirements were calculated. To record their food, dish and beverage intake, users could speak or type in the name of the food, dish or beverage. When users spoke food, dish or beverage names, the application showed its name and the amount of one serving size on the screen. Users could check whether their voice was well recognized accordingly. If not, users could speak again or type in the name of the food, dish or beverage. Users then recorded the proportion of pre-specified portion size (e.g., 1 serving size of bowl). Daily energy and nutrient intakes were calculated by summing the nutrient content for quantities of foods and beverages consumed, which were calculated by multiplying grams of one pre-specified portion size by the proportion of portion size that they recorded. We used the database of National Rural Living Science Institute [11] for grams of one serving size commonly consumed in Korea. Users were instructed to take a photograph to remind them of foods, dishes, and beverages that they consumed on that day, so that they could record the meal later when they had time for data input. Also, Diet-A had a pop-up function that reminded users to record their meals if they did not record their breakfast, lunch or dinner until 11:00 a.m., 3:00 p.m. or 8:00 p.m., respectively. Diet-A provided real-time feedback on intakes of total energy, carbohydrates, protein, fat, saturated fat, sodium, calcium and iron based on the Dietary Reference Intakes for Koreans 2010 [12]. For energy intake, we compared individuals’ energy intake with Estimated Energy Requirements of Dietary Reference Intakes for Koreans 2010 [12]; estimated energy requirement = α + β × age (years) + PA[γ × weight (kg) + δ × height (m)], α: age-and-sex-specific constant, β: age-and-sex-specific coefficient for age, γ: age-and-sex-specific coefficient for weight, δ: age-and-sex-specific coefficient for height, and PA: coefficient for physical activity. Percent energy intake each from carbohydrate, fat and saturated fat was compared to the Acceptable Macronutrient Distribution Range. Intakes of sodium, calcium and iron were compared to intake goal or recommended intakes (RI) of Dietary Reference Intakes for Koreans 2010. On the feedback screen, intakes of total energy, carbohydrates, protein and fat and contribution of each macronutrient to energy were displayed. To build our database of foods and dishes, we used the database of Korea’s Ministry of Food and Drug Safety [13], the food composition table of the National Rural Living Science Institute [8], KNHANES 24-h recall data and nutrient content provided by food product companies.

To provide a short message for disease prevention, we chose four major metabolic disorders—obesity, diabetes, hypertension and dyslipidemia—which are increasing in prevalence in Korea and are the major chronic disorders regularly reported by Korea Health Statistics. The Korea Health Statistics 2013: Korean National Health and Nutrition Examination Survey (KNHANES VI-1) reported that the prevalence of obesity (body mass index, BMI ≥ 25 kg/m^2^), diabetes, hypertension, hypercholesterolemia and hypertriglyceridemia among Korean adults 30 years or older was 32.5%, 11.9%, 30.4%, 15.9% and 17.6%, respectively [14]. We selected four unfavorable nutrients: total energy intake for obesity, refined carbohydrates for diabetes, sodium for hypertension and saturated fat for dyslipidemia. Diet-A displayed the number of days these nutrients were consumed at levels above the reference over the last 7 days; if the number of days from 4 to 5, Diet-A alerted users of “high” disease risk, and if it ranged from 6 to 7 days, “very high”. Additionally, the percentage of these nutrients relative to their recommended intake and foods or dishes that contributed that nutrient were displayed. Diet-A recommended five foods that contained low amounts of the aforementioned unfavorable nutrients, which were randomly selected in the same food groups for choices for the next meal. 

### 2.2. Intervention Study

#### 2.2.1. Participants

**Figure 2:**
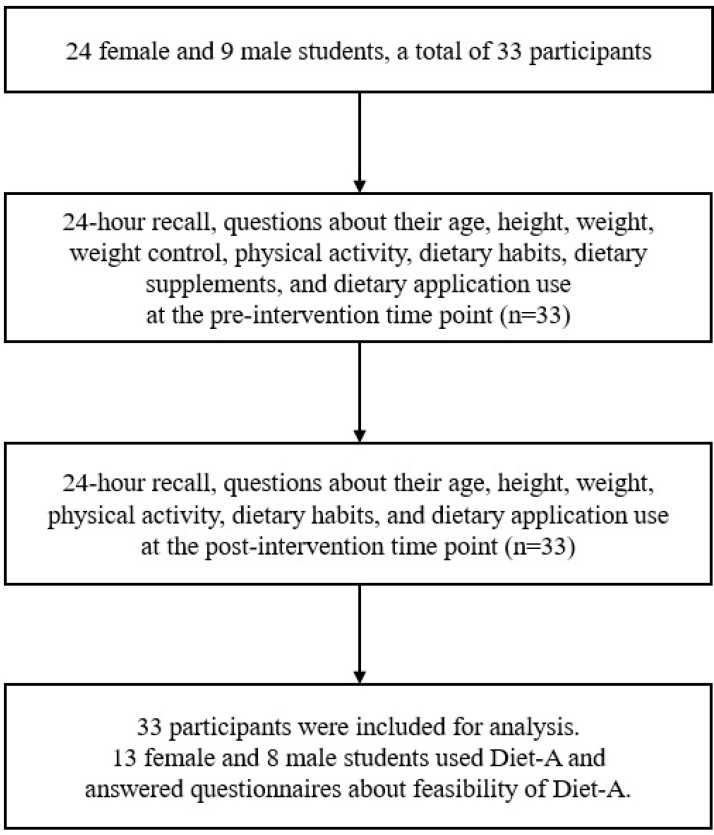
Flowchart of participants.

We recruited male and female high school students from one girls’ high school and one boys’ high school in Seoul, Korea. Eligibility criteria for this study were students who used a smartphone operating on Android and were willing to join. Twenty-four female and nine male students participated in this study. Among the 33 adolescents who met eligibility criteria, 13 female and 8 male students used the mobile application and reported on the feasibility (Figure 2). All 33 participants answered to pre- and post-intervention one-day 24-h recalls and dietary habit questionnaires. All procedures were approved by the Sookmyung Women’s University Institutional Review Board. Informed consent was obtained from the adolescent participants and their parents.

#### 2.2.2. Questionnaires

In the first session, a researcher explained the framework of the study and offered an explanatory note to participants. Participants answered the questions about their age, height, weight, weight control, physical activity, dietary habits, dietary supplements, and dietary application use at the pre-intervention time point. Participants were asked about number of days that participants engaged in vigorous physical activities such as jogging, climbing and cycling at fast speeds for at least 20 min at a time, during the last 7 days. Height and weight were self-reported, and body mass index (BMI) was calculated as body weight (kg) divided by the square of the height (m). Twenty-four-hour recalls were conducted by 5 to 6 trained interviewers at pre-intervention. After the researcher described the functions and how to use the application, participants downloaded the application on their mobile phones. Participants were instructed to record their dietary intake using Diet-A for three months and to record all the foods, dishes, and beverages that they consumed on that day, if they started recording their diet. We also asked interim questions about good and bad aspects of Diet-A and the reasons. 

At post-intervention, participants completed questionnaires about the feasibility and usability of the application. The items on the feasibility questionnaire were adopted from previous feasibility studies of mobile diet applications [15,16,17,18]. Using a five-point Likert scale from 1 (totally disagree) to 5 (totally agree), twenty-one items were designed to measure the degree of satisfaction, convenience and efficiency. Dietary intake post-intervention was assessed using 24-h recalls by trained interviewers. We calculated energy and nutrient intakes of foods and beverages obtained from 24-h recalls using the CAN-Pro 4.0 program [19].

### 2.3. Statistical Analysis

The demographic characteristics between male and female participants were compared using Fisher’s exact test for categorical variables and the Wilcoxon rank-sum test for continuous variables. A paired t-test was used to compare the differences in estimated dietary intake between the two 24-h recalls and Diet-A. Nutrient intake from pre- and post-24-h recalls was compared using paired *t*-test. All statistical analyses in our study were performed using the SAS software package, version 9.4 (SAS Institute Inc., Cary, NC, USA), and all tests were evaluated using two-tailed tests of significance at the *p* < 0.05 level.

## 3. Results

### 3.1. Characteristics of Participants

The demographic characteristics of all participants are shown in Table 1. Nine male and twenty-four female students participated in this study. The mean ages were 16.9 years (range: 16–17 years old) for male students and 17.4 years (range: 16–18 years old) for female students (*p* = 0.016). The mean BMIs were similar between male and female students. Among all participants, 8 male (88.9%) and 13 female students (54.2%) used the mobile application, Diet-A, in the study period. Dietary intake was recorded by Diet-A for an average of 12.2 days (range: 1–47 days) per participant with at least 1 meal per day and 87.9% of participants recorded at least 2 meals per day during the 3-month intervention period (Supplemental Table S1).

### 3.2. Mean Nutrient Intake Estimated by Diet-A and 24-h Recalls

Mean nutrient intakes estimated by the Diet-A and 24-h recalls are shown in Table 2. When we compared the mean values of pre- and post-24-h recalls with Diet-A, the differences between the two methods were statistically significant in energy, carbohydrates, protein, fat, sodium and calcium. The nutrient intakes estimated by Diet-A were lower than those obtained from 24-h recalls. There was no significant difference except for fat and calcium in the male group. However, the results in the female group were similar to those of the total group.

### 3.3. Comparison of the Mean Nutrient Intake Between the Pre- and Post-24-h Recalls

The mean values of energy and nutrient intakes estimated by pre- and post-intervention 24-h recalls were comparable, as shown in Table 3. In the 24-h recall after the intervention, there were statistically significant decreases in sodium (*p* = 0.040) and calcium (*p* = 0.034) compared with the 24-h recall before the intervention. For other nutrients, we did not find significant differences between pre- and post-intervention. In the 24-h recall after the intervention, one male and one female student reported that they suffered from gastritis and enteritis, and one of the girls reported that she had tried a one-meal-a-day diet. When we conducted a sensitivity analysis by excluding these three participants, we did not observe significant differences in any of the nutrients between pre- and post-intervention 24-h recalls. 

On the interim questionnaire for compliance with Diet-A, we asked what good and bad things they found in using the mobile application (*n* = 9). They reported that they were able to get information about their dietary habits and eat meals at regular times. In addition, they believed that they increased their intake of vegetables and fruits and reduced their intake of fast food such as hamburgers or fried chicken during the intervention study. The participants also reported that the main reasons why they stopped using Diet-A were the burden of recording their meals every time they ate and a lack of some foods, such as new commercial products, in the database.

### 3.4. Feasibility of Diet-A

The feasibility results are presented in Table 4. Of those who completed questionnaires (*n* = 21), 61.9% reported that they were satisfied using the application to monitor their dietary intake, and 65.0% of participants who answered in item 8 reported that this application was helpful for monitoring the food consumed. Additionally, 57.1% of participants slightly agreed or totally agreed that they were able to learn about their dietary intake during the period they used a Diet-A. However, when they were asked about the difficulties of application use, 71.4% reported that it was burdensome to record their diet and sometimes they did not remember to record their diet (85.7%).

## 4. Discussion

We developed a mobile application called ‘Diet-A’ that was designed to record dietary intake and provide users with real-time feedback. Diet-A provided real-time feedback on selected nutrients relative to the Korea Dietary Reference Intakes and a short message focusing on the prevention of obesity, diabetes, hypertension and dyslipidemia.

We selected total energy intake for obesity, refined carbohydrate intake for diabetes, sodium intake for hypertension and saturated fat for dyslipidemia based on the following evidence. A long-term excess of energy intake leads to obesity by altering the balance between energy intake and expenditure, and maintaining energy balance is a key strategy in the management of obesity [20]. For prevention of type 2 diabetes, the American Diabetes Association recommended that individuals choose whole-grain foods whenever possible instead of refined foods [21]. Several epidemiologic studies found a positive association between diabetes mellitus and refined grains, including in Asian populations [22]. Excess sodium intake leads to hypertension by increasing blood volume and peripheral vascular resistance. Dietary Approaches to Stop Hypertension (DASH) clinical trials have shown that sodium reduction lowers systolic and diastolic blood pressure levels [23]. Sodium intake is high in Koreans; according to KNHANES in 2013, 80.6% of people over 9 years old consumed more sodium than the 2000 mg/day that the WHO recommends [11]. Therefore, we emphasized sodium intake for hypertension prevention. Saturated fat intake is a risk factor for cardiovascular disease, possibly through modulation of lipid metabolism. A meta-analysis of 60 controlled trials reported that the ratio of total to High-Density Lipoprotein (HDL) cholesterol decreased if saturated fat was replaced with unsaturated fat [24].

Accurate dietary assessment is essential for improving the feasibility of dietary mobile applications. Twenty-four-hour recall, food records, and food frequency questionnaires (FFQs) have been widely used for dietary assessment in epidemiology studies, but none of these methods estimate dietary intake without error [25,26]. Therefore, use of ICT has been suggested to improve dietary assessment [27]. Recent systematic reviews showed a favorable outlook on image-assisted dietary assessment with mobile devices, in that ICT devices could assist the assessment of foods not captured by traditional methods alone [28] with higher user satisfaction and preference than conventional methods [29]. In a study assessing the accuracy of a personal digital assistant (PDA)-based dietary assessment program, Beasley et al. found that use of the PDA decreased the burden on the researcher by removing the need for data entry of nutritional information from the food records [30]. A review of new technology indicated that use of digital methods such as voice recognition or images captured by camera showed promise for enhancing the accuracy of food records [31]. 

During the last few years, clinical trials for managing chronic diseases using ICT have been actively conducted. Use of websites or mobile devices as a self-monitoring system resulted in greater weight loss compared to conventional care in several weight loss intervention studies. In a 12-month randomized clinical trial of 69 obese adults, the PDA group lost over 3.8 kg more than the standard group [32]. David & Rafiullah [33] systematically reviewed the recent clinical studies of the use of mobile health applications for diabetes management and reported that 76% of the 21 papers published from 2007 to 2014 showed a favorable effect on diabetes management. 

Diet-A has features that help individuals improve their dietary habits, including real-time feedback on selected nutrients, disease risk, and food recommendations. Provision of real-time data allows users to read immediate feedback or to get individualized dietary advice. In our study, 65.0% of participants who answered the question reported that this application was the way they monitored their dietary intake, and 61.9% of participants reported satisfaction with realizing their own dietary intake. Although the adolescents in the study favorably answered that they were able to monitor their diet, the present study did not show improvement in their diet when we compared their diet pre- and post-intervention. We selected adolescents in this intervention study because we believed that a mobile application could be a better alternative approach to measure their diet compared to conventional methods because adolescents had more confidence in the use of mobile technology than adults [34,35]. However, nutrient intake estimated by Diet-A was lower than what was estimated by 24-h recalls in all nutrients. Possible explanations may include difficulty in finding time to record in the middle of busy schedules and low motivation for dietary improvement. In our study, 71.4% of participants agreed or totally agreed that the application was burdensome to use, and 85.7% of them reported that sometimes they forgot to record their intakes. Additionally, the limited database of new products could lead to some underestimation. Some studies have shown that adolescents are prone to reporting error because of their unstructured eating patterns and the appearance of altering their food intake to simplify recording [35,36,37]. 

The study has several limitations. First, the sample was small and unrepresentative of the general population. Second, this application did not include a function to measure the user’s physical activity; estimated energy requirements were computed as ‘low-active’, assuming the typical exercise level of Korean high school students. It may be necessary to add a motion sensor function in future studies. We chose only one nutrient per disease for a short message targeting the prevention of metabolic disorders, but given that multiple factors play roles in the development of chronic disease, further studies are needed to implement various components.

## 5. Conclusions

Diet-A is developed to provide users with real-time feedback on their diet and assess food and nutrient intakes. Diet-A is equipped with voice recognition and photography functions for accurate recording. It focuses on four chronic diseases, namely, obesity, diabetes, hypertension, and dyslipidemia, providing users with real-time feedback. However, dietary recording remains burdensome even with the application. Overall, our study revealed some challenges with regard to the use of our mobile application for dietary assessment and self-management of dietary habit, including underestimation, promotion of motivation to use, and improvement of the food and dish database.

## Figures and Tables

**Table 1:** Baseline characteristics of adolescents enrolled in an intervention study of mobile application, Diet-A (*n* = 33).

	Male (*n* = 9)	Female (*n* = 24)	*p* Value ^1^
Characteristics	Mean ± standard deviation		
**Age** (year)	16.9 ± 0.3	17.4 ± 0.6	0.016
**BMI** (kg/m^2^) ^2^	21.4 ± 2.6	22.4 ± 4.5	0.571
	*n* (%)		***p* value** ^3^
**BMI** (kg/m^2^) ^2^			
<18.5	2 (22.2)	2 (8.3)	0.445
18.5 ≤ 23	5 (55.6)	15 (62.5)	
23 ≤ 25	2 (22.2)	3 (12.5)	
25+	0 (0.0)	4 (16.7)	
**Have ever tried to lose weight within 1 year**			
Yes	3 (33.3)	11 (45.8)	0.698
No	6 (66.7)	13 (54.2)	
**Physical activity**			
None	0 (0.0)	16 (66.7)	<0.001
1–2 per week	1 (11.1)	5 (20.8)	
3+ per week	8 (88.9)	3 (12.5)	
**Dietary supplement use**			
Yes	4 (44.4)	7 (29.2)	0.438
No	5 (55.6)	17 (70.8)	

^1^ Differences between male and female were analyzed using Wilcoxon rank sum test. ^2^ Body mass index (BMI), calculated from self-reported weight and height (kg/m^2^). ^3^ Differences between male and female were analyzed using Fisher’s exact test.

**Table 2:** Comparison of the nutrient intake from Diet-A vs. nutrient intake from 24-h recalls among all male and female students (*n* = 21).

Nutrients	Diet-A ^1^ (*n* = 21)	24-h Recalls ^2^ (*n* = 21)	*p* Value ^3^
	mean ± standard deviation		
Energy (kcal/day)	1427 ± 379	1893 ±394	0.002
Carbohydrates (g/day)	198.8 ± 48.8	255.6 ± 54.6	0.003
Protein (g/day)	50.5 ± 19.4	76.2 ± 25.3	0.002
Total fat (g/day)	38.8 ± 15.6	62.0 ± 21.2	<0.001
Sodium (mg/day)	2436.8 ± 956.3	3204.7 ± 1090.6	0.020
Saturated fat (g/day)	11.4 ± 4.0	13.2 ± 8.7	0.390
Calcium (mg/day)	225.4 ± 105.0	511.0 ± 312.1	<0.001
Iron (mg/day)	11.3 ± 6.7	13.5 ± 4.8	0.210

^1^ The average of number of days that participants used Diet-A was 12.2 days (interquartile range: 6–14 days). ^2^ Mean values of two 24-h recalls were estimated by the CAN-Pro 4.0 program. ^3^ Differences between two dietary assessment methods were analyzed using paired *t-*test.

**Table 3:** Comparison of the nutrient intake from pre- and post-24-h recalls among all male and female students (*n* = 33).

Nutrients	24-h Recall at Pre-Intervention (*n* = 33)	24-h Recall at Post-Intervention (*n* = 33)	*p* Value ^1^
	Mean ± standard deviation		
Energy (kcal/day)	1929 ± 668	1696 ± 593	0.107
Carbohydrates (g/day)	261.3 ± 70.9	231.3 ± 87.1	0.068
Protein (g/day)	79.2 ± 46.9	64.8 ± 26.0	0.143
Total fat (g/day)	62.6 ± 33.6	55.1 ± 25.8	0.298
Sodium (mg/day)	3374.5 ± 1869.0	2567.1 ± 1328.8	0.040
Saturated fat (g/day)	10.9 ± 9.2	13.0 ± 9.4	0.280
Calcium (mg/day)	534.6 ± 304.4	390.0 ± 361.0	0.034
Iron (mg/day)	14.6 ± 7.2	11.4 ± 6.3	0.072

There was a 3-month interval between pre-intervention and post-intervention. ^1^ Differences between pre- and post-24-h recalls were analyzed using paired *t-*test.

**Table 4:** Agreement with items on the feasibility questionnaire about Diet-A use among application users (*n* = 21).

Item	*n*	Totally Disagree	Slightly Disagree	Neutral	Slightly Agree	Totally Agree
		*n* (%)				
1. This application was an easy way to monitor my dietary intake	21	1 (4.8)	3 (14.3)	8 (38.1)	6 (28.6)	3 (14.3)
2. I learned about my dietary intake during the period I used Diet-A	21	1 (4.8)	1 (4.8)	7 (33.3)	10 (47.6)	2 (9.5)
3. The function of taking photographs helped me to remember the foods I ate	20	3 (15.0)	3 (15.0)	7 (35.0)	6 (30.0)	1 (5.0)
4. The voice recognizing function helped me to input what I ate in a convenient way	20	5 (25.0)	7 (35.0)	5 (25.0)	3 (15.0)	0 (0.0)
5. I was ashamed to use the voice recognition function	20	3 (15.0)	4 (20.0)	6 (30.0)	4 (20.0)	3 (15.0)
6. The application made me think about how to change my dietary intake	21	1 (4.8)	3 (14.3)	9 (42.9)	6 (28.6)	2 (9.5)
7. This application actually influenced my dietary habits	21	1 (4.8)	5 (23.8)	9 (42.9)	4 (19.1)	2 (9.5)
8. This application was helpful for monitoring the food consumed	20	1 (5.0)	1 (5.0)	5 (25.0)	9 (45.0)	4 (20.0)
9. The application was easy to use	21	3 (14.3)	7 (33.3)	3 (14.3)	4 (19.1)	4 (19.1)
10. I was able to get enough clues about meaning of each menu	21	1 (4.8)	2 (9.5)	10 (47.6)	7 (33.3)	1 (4.8)
11. It was helpful to manage my dietary intake using the application	21	1 (4.8)	3 (14.3)	9 (42.9)	6 (28.6)	2 (9.5)
12. I was able to quickly find the menu that I need from the application	21	4 (19.1)	5 (23.8)	6 (28.6)	4 (19.1)	2 (9.5)
13. The information provided on the application was easy to understand	21	2 (9.5)	5 (23.8)	5 (23.8)	6 (28.6)	3 (14.3)
14. The information provided on the application was helpful	21	1 (4.8)	1 (4.8)	11 (52.4)	7 (33.3)	1 (4.8)
15. I enjoyed using the application	21	2 (9.5)	4 (19.1)	8 (38.1)	6 (28.6)	1 (4.8)
16. I was satisfied with using the application to monitor my dietary intake	21	1 (4.8)	4 (19.1)	3 (14.3)	11 (52.4)	2 (9.5)
17. I liked getting customized information about my dietary intake	21	1 (4.8)	3 (14.3)	7 (33.3)	9 (42.9)	1 (4.8)
18. This application interfered with my daily life	21	6 (28.6)	10 (47.6)	3 (14.3)	1 (4.8)	1 (4.8)
19. It took a long time to use this application	21	2 (9.5)	4 (19.1)	8 (38.1)	6 (28.6)	1 (4.8)
20. It was burdensome to use this application	21	0 (0.0)	3 (14.3)	3 (14.3)	12 (57.1)	3 (14.3)
21. Sometimes, I had trouble remembering to record my dietary intake	21	0 (0.0)	1 (4.8)	2 (9.5)	11 (52.4)	7 (33.3)

## Supplementary Materials

The following are available online at www.mdpi.com/2072-6643/9/7/748/s1, Table S1: The number of days and the number of meals that adolescents recorded in an intervention study of mobile application, Diet-A (*n* = 21).
