# Supplementary material for: Use of a Mobile Application for Self-Monitoring Dietary Intake: Feasibility Test and an Intervention Study
Source: Nutrients. 2017 Jul 13;9(7):748. doi: 10.3390/nu9070748 (PMC5537862; doi:10.3390/nu9070748)
Supplement: Supplementary file 1 [file nutrients-09-00748-s001.zip › nutrients-197967-supplementary.pdf]

Supplemental Table S1. The number of days and the number of meals that adolescents recorded in an intervention study of mobile application, Diet-A (n=21)

	Sex[1]	Number of days recorded[2]	Number of meals recorded		
			1 meal	2 meals	3 meals
Person 1	M	6	1	1	4
Person 2	M	32	0	32	0
Person 3	M	5	1	0	4
Person 4	M	6	3	2	1
Person 5	M	1	0	0	1
Person 6	M	23	0	13	10
Person 7	M	8	0	5	3
Person 8	M	7	3	2	2
Person 9	F	11	0	11	0
Person 10	F	1	0	1	0
Person 11	F	12	1	2	9
Person 12	F	16	2	8	6
Person 13	F	47	1	13	33
Person 14	F	3	1	0	2
Person 15	F	10	5	4	1
Person 16	F	9	5	2	2
Person 17	F	15	2	9	4
Person 18	F	14	2	5	7
Person 19	F	12	3	6	3
Person 20	F	4	1	3	0
Person 21	F	14	0	4	10

[1]M: male student, F: female student

[2]The average was 12.2 days
